# Extracellular vesicles in human fungal pathogens: Biogenesis, functions, and translational applications

**DOI:** 10.71150/jm.2606008

**Published:** 2026-07-31

**Authors:** Catia Mota, Heeyoun Hwang, Hyun Ah Kang

**Affiliations:** 1 Research Unit for Molecular Structure Data and AI, Korea Basic Science Institute, Cheongju 28119, Republic of Korea; 2 Department of Life Science, Chung-Ang University, Seoul 06974, Republic of Korea

**Keywords:** fungal extracellular vesicles, EV biogenesis, bioactive cargo, host-pathogen interactions, immunomodulation

## Abstract

Fungal extracellular vesicles (EVs) have emerged as critical mediators of fungal physiology, virulence, and host–pathogen interactions. Since their first description in *Cryptococcus neoformans*, EVs have been identified in several fungal species and shown to transport a broad repertoire of bioactive cargo. Increasing evidence indicates that fungal EVs participate in multiple biological processes, including cell wall remodeling, stress adaptation, biofilm formation, antifungal resistance, and modulation of host immune responses. Recent advances in cryo-electron microscopy, multi-omics approaches, and functional genetics have substantially expanded our understanding of the molecular mechanisms governing EV biogenesis, cargo selection, and extracellular trafficking. These studies have further revealed that EV cargo loading is a highly regulated process linked to intracellular proteostasis, glycosylation, lipid homeostasis, and environmental adaptation. In parallel, the intrinsic immunogenicity and structural stability of fungal EVs have highlighted their translational potential as diagnostic biomarkers, vaccine platforms, therapeutic targets, and nanoscale delivery systems. Given the increasing global burden of invasive fungal infections, this review focuses on EVs derived from clinically relevant human fungal pathogens. We summarize recent advances in EV biogenesis, cargo regulation, their roles in pathogenesis, highlight emerging translational applications, and discuss key unresolved questions and future research directions in the field.

## General Concepts of Fungal Extracellular Vesicles

Fungal infections constitute a major and underrecognized global health burden. More than one billion individuals are affected by fungal infections annually, and invasive mycoses account for approximately 1.6 million deaths worldwide, particularly among immunocompromised individuals (Almeida et al., 2019; Brown et al., 2012). The increasing prevalence of multidrug-resistant fungi, including *Cryptococcus neoformans*, *Candidozyma auris* (formerly *Candida auris*), *Aspergillus fumigatus*, and *Candida albicans*, has intensified the need to better understand fungal pathogenic mechanisms and develop innovative therapeutic strategies (Casadevall et al., 2019; Denning, 2024; Perfect, 2017; Rajasingham et al., 2022; Zhao et al., 2023). In 2022, the World Health Organization (WHO) designated several fungal pathogens as priority pathogens, classifying four as critical priority because of their high mortality rates and limited treatment options (Fisher and Denning, 2023). Within this global health context, elucidating the mechanisms that regulate fungal communication, adaptation, and virulence has become increasingly important.

Extracellular vesicles (EVs) have emerged as conserved mediators of intercellular communication across all domains of life (Gill et al., 2018). EVs are lipid bilayer-enclosed, non-replicating nanoparticles released by living cells into the extracellular milieu (Rodrigues et al., 2015; van Niel et al., 2018). These vesicles transport diverse bioactive cargo, including proteins, lipids, carbohydrates, metabolites, and nucleic acids, thereby enabling coordinated signaling between cells and across species. The International Society for Extracellular Vesicles (ISEV) recommends describing EV populations based on their physical and biochemical characteristics, including sedimentation properties (Mateescu et al., 2017; Théry et al., 2018). Nevertheless, many studies continue to use traditional nomenclature based on biogenesis and size, categorizing EVs as exosomes, which are typically 30–150 nm in diameter and of endosomal origin; microvesicles or ectosomes, which are 300–1000 nm in diameter and formed by plasma membrane (PM) budding; and apoptotic bodies, which are > 1 μm in diameter (Fig. 1A) (Gill et al., 2018; Liebana-Jordan et al., 2021). Throughout this review, the terms “exosome-like EVs” and “microvesicle-like EVs” are used when referring studies that infer specific biogenetic pathways. However, because the precise subcellular origins of fungal EV subpopulations remain unresolved and isolation procedures typically recover heterogeneous vesicle mixtures, the inclusive term “fungal EVs” is used whenever biogenetic origin cannot be definitively assigned.

The discovery of EV production in fungi significantly expanded the conceptual framework of the canonical protein secretion pathway, which depends on a defined membrane-bound route through the endoplasmic reticulum (ER), Golgi apparatus, and PM (Lee et al., 2004; Schekman, 2002). Fungal EVs were formally characterized in 2007, when Rodrigues and colleagues demonstrated that *C. neoformans* secretes membrane-bound vesicles containing the capsular polysaccharide glucuronoxylomannan (GXM) that traverses the cell wall (Rodrigues et al., 2007). This finding fundamentally challenged the long-standing assumption that the dense fungal cell wall precludes vesicular export. Since then, EV production has been described in a wide range of yeasts and filamentous fungi (Table 1), including clinically relevant pathogens such as *C. neoformans*, *C. albicans*, *C. auris*, *Cryptococcus gattii*, *Histoplasma capsulatum*, *A. fumigatus*, *Paracoccidioides brasiliensis*, *Sporothrix brasiliensis*, and *Malassezia sympodialis*, indicating that vesicular secretion is conserved across phylogenetically diverse fungal species (Albuquerque et al., 2008; Bielska et al., 2018; Botbol et al., 2026; Gehrmann et al., 2011; Ikeda et al., 2018; Oliveira et al., 2010b; Reis et al., 2021; Rizzo et al., 2020; Souza et al., 2019; Vallejo et al., 2011; Zamith-Miranda et al., 2021a).

Functionally, fungal EVs are now recognized as dynamic regulators of fungal physiology and pathogenicity. In addition to their major role in unconventional protein secretion, EVs have been implicated in the horizontal transfer of virulence traits, biofilm matrix assembly, regulation of morphological transitions, prion-like protein propagation, and modulation of antifungal tolerance and resistance (Bielska et al., 2018; Chan et al., 2022; Honorato et al., 2022; Rizzo et al., 2023). In pathogenic contexts, EVs deliver virulence determinants and immunomodulatory molecules, thereby shaping host–pathogen interactions and influencing infection outcomes (Bielska et al., 2018; Oliveira et al., 2010b; Vargas et al., 2015). These findings position fungal EVs not merely as passive carriers of cellular material, but as active modulators of stress adaptation, communication, and disease progression.

Several recent reviews have summarized individual aspects of fungal EV biology, including vesicle biogenesis, cargo composition, and host–pathogen interactions. However, a comprehensive review integrating these aspects while incorporating recently uncovered mechanistic insights and emerging translational applications across clinically relevant human fungal pathogens is still lacking. In this review, we focus on EVs derived from important medical fungal pathogens, prioritizing those included in the WHO fungal priority pathogens list (Fisher and Denning, 2023), particularly the four fungal species, *C. neoformans*, *C. auris*, *A. fumigatus*, and *C. albicans*, which are designated as critical priority pathogens. We also discuss EVs from additional fungal species for which notable findings have been reported (Table 1). Furthermore, we examine how advances in the understating of EV biology in human fungal pathogens are informing the development of diagnostic, therapeutic, and vaccine strategies, while highlighting the major challenges that currently hinder clinical translation.

## Biogenesis and Cargo of Fungal Extracellular Vesicles

### EV biogenesis pathways

The molecular mechanisms underlying fungal EV biogenesis remain incompletely characterized. Nevertheless, advances in biophysical and high-resolution imaging approaches have substantially improved the structural characterization of fungal EV populations. Nanoparticle tracking analysis (NTA) has revealed broad and often multimodal vesicle size distributions, supporting the coexistence of distinct EV subpopulations that differ in size and likely arise through different biogenetic routes (Fig. 1B) (Mota et al., 2025; Reis et al., 2021; Welsh et al., 2024). Complementary cryogenic electron microscopy (cryo-EM) based studies have further demonstrated considerable morphological heterogeneity, including spherical, multilayered, and electron-dense vesicles with diverse membrane architectures (Fig. 1C) (Mota et al., 2025; Reis et al., 2021; Rizzo et al., 2021). Together, these findings support the concept that fungal EVs comprise structurally diverse extracellular particles generated through multiple intracellular pathways, paralleling the mechanistic diversity described in mammalian EV systems. Vesicles are generated at distinct subcellular sites and likely originate through multiple biogenetic routes, including endosomal multivesicular body (MVB)-associated pathways and PM budding mechanisms, which selectively recruit components involved in vesicle trafficking, membrane remodeling, and unconventional secretion (Rodrigues et al., 2011; Wolf et al., 2014).

#### 1. Endosomal and multivesicular body (MVB)-associated pathways

A central mechanism proposed for EV biogenesis involves the endosomal system. In eukaryotic cells, inward budding of endosomal membranes generates intraluminal vesicles (ILVs) within MVBs, which subsequently fuse with the PM to release their luminal contents extracellularly as exosome-like particles (Fig. 1A, left) (Oliveira et al., 2010b; Raposo and Stoorvogel, 2013; Rodrigues et al., 2011). MVB formation depends on the endosomal sorting complex required for transport (ESCRT) machinery, which mediates cargo recognition, membrane deformation, and vesicle scission through the sequential assembly of ESCRT-0, -I, -II, and -III complexes (Henne et al., 2011; Williams and Urbé, 2007). The process is initiated by phosphorylation of phosphatidylinositol to phosphatidylinositol 3-phosphate on the endosomal membrane by the phosphatidylinositol 3-kinase Vps34, which recruits ESCRT-0 components and subsequently triggers the downstream complexes required for ILV formation and MVB maturation (Henne et al., 2011; Williams and Urbé, 2007). Genetic evidence has established the relevance of this pathway in fungi. In *C. albicans*, the deletion of ESCRT-associated genes substantially reduced EV output (Zarnowski et al., 2018). Similarly, in *C. neoformans*, loss of Vps27, a core ESCRT-0 component, resulted in abnormal vesicle trafficking and intracellular accumulation of MVBs (Park et al., 2020). Additional ESCRT mutants, including *vps23*Δ and *snf7*Δ, exhibited defective secretion of EV-associated virulence factors and attenuated virulence in murine cryptococcosis models (Godinho et al., 2014; Hu et al., 2013, 2015; Park et al., 2020). Collectively, these findings underscore the central role of ESCRT-mediated trafficking in fungal EV biogenesis and pathogenicity. Interestingly, ESCRT-independent exosome biogenesis has also been reported as an alternative mechanism for ILV formation in mammalian systems (Stuffers et al., 2009).

Several protein components associated with Golgi organization and post-Golgi trafficking also contribute to EV formation. The Golgi reassembly and stacking protein (GRASP), localized at the trans-ER/Golgi interface, is required for unconventional protein secretion and EV biogenesis, while indirectly influencing conventional secretion through its role in Golgi organization (Kinseth et al., 2007; Kmetzsch et al., 2011; Peres da Silva et al., 2018). In *C. neoformans*, GRASP deletion altered EV size distribution and cargo composition and reduced virulence (Kmetzsch et al., 2011; Peres da Silva et al., 2018). Increasing evidence also supports the participation of conventional post-Golgi secretory machinery in fungal EV biogenesis. Among these components, Sec6, an essential component of the exocyst complex that mediates tethering and fusion of post-Golgi secretory vesicles at the PM, has emerged as a critical modulator of EV biogenesis. In *C. neoformans*, RNA interference (RNAi)-mediated knockdown of *SEC6* significantly reduced EV secretion, establishing a direct mechanistic link between conventional exocytotic machinery and EV release (Panepinto et al., 2009). Similarly, Sec1, another regulator of Golgi-derived vesicle fusion at the PM, selectively influences EV cargo composition without affecting overall vesicle release, suggesting that EV cargo loading and vesicle production are subject to distinct regulatory mechanisms (Oliveira et al., 2010b). Sec4, a Rab family GTPase required for vesicle-mediated exocytosis and autophagy, additionally affects both EV composition and extracellular release kinetics (Oliveira et al., 2010b). Together, these observations support a close functional relationship between fungal EV biogenesis and the highly regulated vesicle trafficking and membrane fusion events that coordinate the conventional secretion pathway.

#### 2. Plasma membrane budding

An alternative mechanism of EV biogenesis involves direct outward budding from the PM, yielding microvesicle-like particles, also referred to as ectosomes (Fig. 1A). Electron microscopy-based studies have documented morphological evidence of vesicular protrusions emerging from the PM across multiple fungal species, including *C. neoformans*, *C. albicans*, and *A. fumigatus* (Rizzo et al., 2020, 2021; Rodrigues et al., 2008; Wolf et al., 2015). Vesicles generated through PM budding (300–1000 nm) are generally larger than those originating from endosomal MVBs (30–150 nm). This process appears to be facilitated by localized membrane curvature induced by lipid microdomains enriched in ergosterol and sphingolipids, which modulate membrane fluidity and budding dynamics (Reis et al., 2019; Vallejo et al., 2012; Wolf et al., 2015).

The molecular mechanisms of microvesicle biogenesis are less well characterized than of those underlying endosomal EV formation. In mammalian systems, PM shedding is regulated by small GTPases, including ARF6 and Rho family proteins, which coordinate actin cytoskeleton remodeling and generate the contractile forces required for vesicle scission through actin–myosin interactions (Tricarico et al., 2017). Fungal homologs of these regulators, including Rho-type GTPases (Rho1, Rho3, Rac1, and Cdc42) and Arf3, the fungal counterpart of mammalian ARF6, have been identified in fungal EV proteomes and are likely involved in analogous membrane remodeling processes during vesicle release (Dawson et al., 2020). In addition to cytoskeleton-associated regulators, components of the ESCRT machinery also contribute to PM-derived EV formation. ESCRT-III components and the ATPase Vps4 participate in membrane remodeling and scission events required for ectosome biogenesis, and *C. albicans* mutants lacking multiple ESCRT orthologs exhibit markedly reduced EV production (Zarnowski et al., 2018). These observations indicate that PM budding and endosomal EV biogenesis are not fully independent processes, but are rather interconnected pathways that share components of the broader cellular membrane trafficking machinery.

Despite this interconnection, several studies support the existence of mechanistically distinct EV subpopulations within the same fungal species. In *C. albicans*, yeast-derived EVs are generally larger and enriched in cell wall proteins, consistent with a PM-budding origin, whereas hypha-derived EVs are smaller and enriched in ESCRT-associated proteins, supporting an endosomal origin (Martínez-López et al., 2022). Lipidomic analyses have further reinforced the existence of pathway-specific biogenesis mechanisms. EVs from *P. brasiliensis* display sterol and fatty acid profiles distinct from those of whole cells, with additional isolate-specific differences, suggesting selective membrane sourcing during vesicle formation (Vallejo et al., 2012).

#### 3. Apoptotic bodies

Dying fungal cells can generate larger extracellular vesicular structures referred to as apoptotic bodies (Fig. 1A, right) (Gill et al., 2018; Liebana-Jordan et al., 2021). Unlike EVs released through active secretory processes, apoptotic bodies arise from PM blebbing and cellular fragmentation during programmed cell death or severe cellular stress. Apoptotic bodies are generally larger and more heterogeneous than conventional fungal EVs, frequently exceeding 1 μm in diameter, and may contain cytoplasmic material, organelle fragments, nucleic acids, and membrane-associated proteins derived from disassembling cells (Atkin-Smith et al., 2015; Raposo and Stoorvogel, 2013). In contrast to the selective cargo loading observed in exosome-like vesicles and microvesicles, the molecular composition of apoptotic bodies largely reflects the passive incorporation of intracellular material during cellular disassembly.

Although apoptotic body formation has been extensively characterized in mammalian systems, its contribution to fungal extracellular vesicle biology remains poorly understood. Nevertheless, apoptotic vesicle-like structures have been observed in fungal cultures exposed to environmental stress and antifungal treatment, suggesting that programmed cell death and cellular damage contribute to the heterogeneous extracellular particle populations recovered from fungal supernatants (Carmona-Gutierrez et al., 2018; Rodrigues et al., 2015). Because apoptotic bodies may contain intracellular material passively released during cell fragmentation, distinguishing these structures from actively secreted EV populations remains a major technical challenge in fungal EV isolation and characterization studies.

Autophagy-associated pathways have been implicated in fungal EV biology, although via mechanisms distinct from apoptotic body formation. In *C. neoformans*, deletion of the autophagy regulator *ATG7* resulted in hypovirulence and altered EV RNA composition compared with wild-type strains, supporting a functional connection between autophagy-related pathways and vesicle-mediated secretion (Oliveira et al., 2016; Peres da Silva et al., 2018). These findings highlight the considerable mechanistic and structural heterogeneity of fungal extracellular particles and emphasize the importance of distinguishing actively secreted EVs from vesicles generated during cellular stress, autophagy, or apoptotic disassembly.

### EV trafficking across the fungal cell wall

A defining feature of fungal EV biology is the requirement for vesicle transit across the cell wall, a multilayered matrix composed primarily of chitin, β-glucans, and mannoproteins. Unlike mammalian cells, which release EVs directly into the extracellular space, fungi must export vesicles through this dense structural barrier. This process extends beyond a simple biophysical challenge as EV passage can actively reshape wall composition and surface antigen exposure, thereby influencing host-pathogen interactions and immune recognition.

Multiple non-mutually exclusive models have emerged to explain EV transit across the fungal cell wall. One proposed mechanism involves turgor-driven extrusion, whereby vesicle accumulation in the periplasmic space generates mechanical pressure that forces vesicles through naturally existing pores within the cell wall matrix (Rodrigues et al., 2015; Wolf et al., 2014). A second mechanism involves enzyme-assisted remodeling, in which EV-associated enzymes, including glycan hydrolases such as β-glucosidases and endochitinases, locally degrade structural polysaccharides to facilitate vesicle passage (Albuquerque et al., 2008; Rodrigues et al., 2007, 2008; Vallejo et al., 2011). A third model suggests that EVs traverse pre-existing pore channels, potentially undergoing deformation to adapt to pore morphology, potentially aided by cytoskeleton-dependent processes (Albuquerque et al., 2008; Rodrigues et al., 2008, 2015).

Proteomic characterization of EVs from *C. albicans*, *H. capsulatum*, and *P. brasiliensis* provide substantial support for the enzyme-mediated remodeling hypothesis (Albuquerque et al., 2008; Gil-Bona et al., 2015; Rodrigues et al., 2007, 2008; Vargas et al., 2015). Fungal EV cargo consistently includes cell wall-degrading enzymes and hydrolases that are capable of modifying the local wall environment during stress responses or morphological transitions. Consequently, EV release and wall transit may represent a mechanism for rapid surface remodeling that enables fungi to modify wall architecture in response to environmental changes, with implications for host-pathogen interactions (Ene et al., 2015; Erwig and Gow, 2016; Fischer et al., 2008; Free, 2013).

### Molecular cargo of fungal EVs

Fungal EVs contain a complex and heterogeneous repertoire of biomolecules, including proteins, lipids, polysaccharides, nucleic acids, and metabolites (Fig. 1D). These components collectively participate in diverse biological processes such as cell wall remodeling, stress responses, metabolic coordination, intercellular communication, and host–pathogen interaction (Albuquerque et al., 2008; Oliveira et al., 2010b; Rodrigues et al., 2008; Vargas et al., 2015). Importantly, EV cargo composition does not merely reflect whole-cell content. Instead, specific functional classes of molecules are selectively enriched within vesicles, suggesting that cargo incorporation into vesicles is at least partially regulated during EV biogenesis rather than representing a random sampling of cytoplasmic content.

#### 1. Protein cargo

Proteins represent the most extensively characterized components of fungal EVs. Across fungal species, EV proteomes consistently include several functional categories, such as metabolic enzymes, stress-response proteins, virulence-associated factors, cell wall–remodeling enzymes, and membrane-associated transport proteins. EV cargo proteins include both classical secretory proteins containing signal peptides and proteins lacking conventional secretion signals, emphasizing the role of EVs in unconventional protein export.

Proteomic analyses of cryptococcal EVs have identified multiple cell surface glycoproteins, including members of the chitin deacetylase (CDA) family, several mannoproteins, and proteins belonging to the Tsh, Gox, and Ril families (Mota et al., 2025; Rizzo et al., 2021; Rodrigues et al., 2008; Wolf et al., 2014). EVs are additionally enriched in virulence-associated enzymes such as laccase and urease, relative to their abundance in whole-cell lysates, supporting the existence of selective cargo enrichment mechanisms (Mota et al., 2025; Rizzo et al., 2021; Rodrigues et al., 2007, 2008; Wolf et al., 2014). Recent structural and functional studies further demonstrated that *O*-mannosylation critically influences the stability, proteolytic processing, and secretion of the glycosylphosphatidylinositol (GPI)-anchored mannoproteins Mp88 and Cda1 in *C. neoformans*, emphasizing the importance of glycan architecture in shaping the extracellular fate and immunological properties of EV-associated mannoproteins (Thak et al., 2025). Consistent with these findings, cryo-EM-based analyses revealed that the majority of cryptococcal EVs are surrounded by a distinctive fibrillar surface decoration composed predominantly of mannoproteins, reinforcing that glycosylated surface components represent major structural and functional determinants of cryptococcal EVs (Mota et al., 2025; Rizzo et al., 2021).

Similar complexity exists in the EV proteome of other pathogenic fungi. In *C. albicans*, EVs contain proteins involved in cell wall remodeling, hyphal morphogenesis, biofilm formation, stress adaptation, and antifungal resistance, including Sur7 family members and azole-resistance transporters Cdr1 and Cdr2 (Dawson et al., 2020; Gil-Bona et al., 2015; Holmes et al., 2008; McKenna et al., 2023). Comparative proteomic studies demonstrated that EV composition differs according to fungal morphology, with hyphal EVs displaying a more diverse proteome enriched in ESCRT-associated proteins and intracellular trafficking components, whereas yeast-derived EVs are enriched in cell wall–associated proteins involved in cell wall maintenance (Martínez-López et al., 2022).

EVs from *H. capsulatum* carry antioxidant enzymes such as superoxide dismutase and catalase B which contribute to fungal defense against host-derived oxidative stress. These vesicles also carry carbohydrate-active enzymes, including glucanases and endochitinases, involved in cell wall remodeling (Albuquerque et al., 2008). Comparative proteomic analysis of *H. capsulatum* EVs and whole-cell lysates further demonstrated substantial compositional divergence, providing strong evidence for selective cargo loading during EV biogenesis (Zamith-Miranda et al., 2021b).

EV proteome of *P. brasiliensis* represents an enriched, defined subset of the extracellular proteome associated with metabolism, signaling, and transport functions (Vallejo et al., 2011). Similarly, EVs derived from *A. fumigatus* protoplasts are enriched in proteins associated with lipid metabolism, cell wall biosynthesis, and pathogenicity (Rizzo et al., 2020). EVs from *Candidozyma haemulonii* var. *vulnera* are also enriched in proteins associated with metabolism, proteostasis, and cellular regulation, including the 14-3-23 protein Bmh1, the elongation factor Tef1, and key glycolytic enzymes, such as Cdc19 (pyruvate kinase) and Pdc11 (pyruvate decarboxylase). The additional enrichment of proteasome complex subunits further suggests that EVs may contribute to protein quality control and cellular remodeling (Oliveira et al., 2025).

Collectively, these findings reinforce the concept that fungal EVs are highly specialized and dynamically regulated compartments whose protein cargo composition reflects species-specific biology, environmental adaptation, and pathogenic potential.

#### 2. Lipids

Lipids constitute another major class of EV cargo and play essential roles in vesicle structure and function. Fungal EV membranes are enriched in sterols, phospholipids, and sphingolipids, including glucosylceramides (GlcCers), which are fundamental PM components (Albuquerque et al., 2008; Rizzo et al., 2021; Rodrigues et al., 2007; Vallejo et al., 2012). These lipids contribute to membrane curvature, stability, and vesicle fusion properties. Common phospholipid species in fungal EVs include phosphatidylcholine (PC), phosphatidylserine (PS), and phosphatidylethanolamine (PE) (Albuquerque et al., 2008; Rodrigues et al., 2007). EVs from *H. capsulatum* are markedly depleted in energy storage lipids, such as triacylglycerol, and mitochondrial lipids, such as cardiolipin, compared with whole cells, indicating selective biogenesis processes rather than passive membrane shedding (Zamith-Miranda et al., 2021b).

Lipid composition varies among species, reflecting differences in sterol and sphingolipid metabolism. For instance, EVs produced by *C. albicans* contain higher concentrations of ergosterol than those derived from *C. auris* (Vargas et al., 2015; Zamith-Miranda et al., 2021a). In contrast, *C. auris* EVs are enriched in neutral lipids, particularly diacylglycerols and triacylglycerols, suggesting species-specific lipid packaging strategies that may influence membrane properties, vesicle stability, and host interactions (Zamith-Miranda et al., 2021a). Such compositional differences may contribute to the distinct pathogenic traits and environmental resilience associated with *C. auris*.

Although direct lipidomic characterization remains limited for many fungal species, recent EV proteomic analysis of the emerging pathogen *C. haemulonii* revealed a distinct functional cluster of fatty acid metabolism enzymes among exclusively packaged proteins (Oliveira et al., 2025). This suggests that EVs may possess active lipid remodeling capacity beyond static cargo transport, potentially enabling dynamic membrane modifications and adaptation to host environments after secretion. The selective packaging of these metabolic enzymes indicates species-specific strategies for lipid homeostasis within EVs, although comprehensive lipid profiling of the *C. haemulonii* species complex remains to be conducted. Such metabolic versatility within EVs represents an underexplored mechanism that may contribute to the enhanced environmental resilience and multidrug-resistance phenotypes observed in this emerging pathogen.

#### 3. Polysaccharides and glycoconjugates

Polysaccharides and glycoconjugates constitute major structural and functional components of fungal EVs. Their composition varies considerably among fungal species, reflecting differences in cell wall architecture, capsule biosynthesis, and glycosylation pathways. Comparative analyses across *Cryptococcus* species, including *C. neoformans*, *C. deneoformans*, and *C. deuterogattii*, reveal a conserved core repertoire of EV-associated glycoconjugates and mannoproteins, accompanied by species-specific variations in carbohydrate composition and abundance that likely contribute to niche adaptation and virulence (Bielska et al., 2018; Rizzo et al., 2021). As encapsulated fungi, *Cryptococcus* species additionally package GXM, the major polysaccharide component of the capsule, within EVs (Mota et al., 2025; Rizzo et al., 2021; Rodrigues et al., 2007).

Cell wall-derived polysaccharides are also prominent EV constituents in non-encapsulated fungi. EVs from *H. capsulatum* contain α-glucans and β-glucans enriched relative to whole-cell preparations (Albuquerque et al., 2008), whereas *P. brasiliensis* EVs carry β-glucans and galactose-containing glycans that exhibit selective enrichment compared with cellular carbohydrate pools (Peres da Silva et al., 2015a; Vallejo et al., 2012). In *C. albicans*, biofilm-derived EVs are enriched in mannan and β-glucan, the major structural polysaccharides of the fungal cell wall, supporting their contribution to extracellular matrix organization (Zarnowski et al., 2018). Likewise, EVs from the multidrug-resistant pathogen *C. auris* contain distinct carbohydrate populations that differ in abundance and composition from those of *C. albicans*, further highlighting the existence of species-specific EV glycomes (Zamith-Miranda et al., 2021a). Additional evidence for selective carbohydrate packaging comes from *A. fumigatus*, where protoplast-derived EVs produced during cell wall regeneration contain galactosaminogalactan (GAG), a virulence-associated extracellular polysaccharide. Notably, GAG-associated N-acetylgalactosamine (GalNAc) residues are absent from EVs released by non-regenerating protoplasts, suggesting that EVs contribute directly to polysaccharide export and surface assembly during active cell wall biosynthesis (Rizzo et al., 2020).

Glycoconjugates, including heavily glycosylated membrane-associated proteins, represent another major component of fungal EVs. In cryptococcal EVs, mannoproteins contribute to the formation of the characteristic fibrillar surface layer and account for a substantial proportion of EV-associated carbohydrate content. Bioinformatics analysis of the EV-associated protein sequences in *Cryptococcus* species suggested that many of them are membrane bound proteins bearing at least one putative transmembrane domain or putative GPI-anchor motif and subject to extensive glycosylation (Mota et al., 2025; Rizzo et al., 2021; Thak et al., 2025). Similar glycosylated surface molecules have been identified in EVs from *C. albicans* and other pathogenic fungi, supporting the view that glycoconjugates are integral structural components of fungal EVs rather than merely protein cargo carrying incidental glycan modifications (Vargas et al., 2015).

The polysaccharide and glycoconjugate composition of fungal EVs is highly dynamic and responsive to environmental conditions. Nutrient availability alters EV-associated glycan profiles in *H. capsulatum* (Cleare et al., 2020), antifungal exposure remodels carbohydrate cargo in *C. auris* (Amatuzzi et al., 2022), and biofilm growth promotes the accumulation of matrix-associated polysaccharides in *C. albicans* EVs (Honorato et al., 2022; Zarnowski et al., 2018). Altogether, these observations indicate that EV-associated carbohydrate and glycoconjugate cargo is tightly linked to fungal physiology and environmental adaptation, supporting specialized roles in cell wall biogenesis, extracellular matrix organization, and host–pathogen interactions.

#### 4. Nucleic acids

Fungal EVs also transport nucleic acids, expanding their functional repertoire beyond protein, carbohydrate, and lipid delivery. Similar to mammalian systems, EVs can mediate the transfer of RNA molecules between cells, supporting a mechanism of intercellular communication (Valadi et al., 2007). The presence of RNA in fungal EVs was first described in *C. neoformans* and later confirmed across multiple fungal species (Alves et al., 2019; Nicola et al., 2009; Peres da Silva et al., 2015b, 2019).

Fungal EVs harbor a diverse array of RNA species, including messenger RNA (mRNA), transfer RNA (tRNA), ribosomal RNA (rRNA), small nuclear RNA (snRNA), long noncoding RNA (lncRNA), and microRNA-like molecules (Alves et al., 2019; Liu et al., 2020; Nicola et al., 2009; Peres da Silva et al., 2015b, 2019; Reis et al., 2019). Across species, EV-associated RNAs are predominantly short (< 250 nucleotides) and enriched in noncoding RNA populations, particularly miRNA-like sequences, antisense RNAs, and tRNA-derived fragments. Additional classes such as small nucleolar RNAs (snoRNAs) and regulatory noncoding RNAs (ncRNAs) have also been identified in *C. auris*, highlighting the extensive diversity of EV RNA cargo (Munhoz da Rocha et al., 2021). The species-specific RNA profiles and differential RNA abundance suggests that EV-associated RNAs contribute to fungal adaptation, virulence, and niche specialization (Alves et al., 2019; Peres da Silva et al., 2015b).

Notably, RNA incorporation into fungal EVs is a selective and regulated process rather than a passive reflection of intracellular RNA abundance. Comparative transcriptomic analyses have revealed marked differences between cellular and EV-associated RNA populations. In *C. auris*, EVs are enriched in small RNAs and ncRNAs, whereas abundant cellular RNAs such as rRNA are underrepresented (Munhoz da Rocha et al., 2021). Similarly, in *H. capsulatum*, EV-associated RNAs show little correlation with cellular transcript levels and are instead enriched in transcripts related to stress responses and translation (Alves et al., 2019). These observations strongly support the existence of active RNA sorting mechanisms during EV biogenesis. Additional evidence for selective and species-specific EV cargo loading comes from *C. gattii*, in which EVs derived from hypervirulent outbreak strains enhance the intracellular proliferation of less virulent neighboring strains within macrophages (Bielska et al., 2018). This phenomenon specifically requires hypervirulent *C. gattii* EVs carrying functional protein and RNA cargo and cannot be replaced by EVs derived from *C. neoformans* or non-outbreak *C. gattii* strains, highlighting that EV-mediated communication depends on highly regulated and species-specific cargo composition.

In *Paracoccidioides* species, EV-associated exonic small RNAs (exonic sRNAs) have been proposed as regulatory molecules capable of interacting with coding and untranslated regions of target mRNAs, thereby modulating gene expression. These RNAs have been linked to the regulation of key biological processes, including metabolism, cell wall biosynthesis, vesicle trafficking, and stress responses (Peres da Silva et al., 2019). In *C. haemulonii*, EVs contain a highly diverse repertoire of miRNA-like molecules, many of which are predicted to target mitochondrial pathways, including electron transport chain components and citric acid cycle enzymes (Oliveira et al., 2025).

Collectively, EV-associated RNAs represent a conserved and functionally diverse component of fungal secretion systems, contributing to intercellular communication, environmental adaptation, and host–pathogen interactions, although their precise mechanistic roles remain to be fully elucidated.

### Regulation of fungal EV biogenesis and cargo loading

In addition to the conventional secretion pathway described above, multiple cellular processes contribute to the regulation of fungal EV biogenesis and cargo composition. Membrane lipid organization represents a key determinant of EV formation. In *C. neoformans*, the lipid flippase Apt1, which maintains membrane phospholipid asymmetry, is required for proper EV biogenesis and fungal virulence (Rizzo et al., 2014, 2018). More recently, deletion of *UGG1*, which encodes UDP-glucose glucosyltransferase, a key sensor of the *N*-glycan-dependent endoplasmic reticulum protein quality control (ERQC) system, was shown to alter EV biogenesis and cargo composition in *C. neoformans*, leading to defective capsule polysaccharide secretion (Mota et al., 2025). Together, these observations demonstrate that fungal EV production is tightly coupled to multiple secretory pathways and overall membrane homeostasis.

Cell wall architecture also appears to directly influence EV trafficking efficiency. In *C. neoformans*, melanization correlates with vesicle accumulation in the periplasmic space and decreased cell wall porosity, demonstrating that structural modifications can alter export efficiency (Eisenman et al., 2009; Jacobson and Ikeda, 2005; Rodrigues et al., 2008; Wolf et al., 2014). Genetic studies provide further evidence that EV release involves regulated cellular processes rather than passive diffusion. Deletion of individual chitin synthase genes produces differential effects on vesicle production, suggesting that EV export requires coordinated regulation of cell wall synthesis and remodeling pathways (Banks et al., 2005; Rodrigues et al., 2018).

Despite extensive characterization of EV cargo composition, the mechanisms governing cargo selection remain incompletely understood. A central unresolved question is whether EV-associated molecules are selectively sorted into vesicles or incorporated passively during membrane remodeling and vesicle formation. Accumulating evidence strongly supports regulated cargo enrichment rather than random encapsulation of cytoplasmic material. In *C. neoformans*, EVs are enriched in virulence-associated proteins, including laccase and urease, relative to their abundance in whole-cell lysates (Mota et al., 2025; Rizzo et al., 2021; Rodrigues et al., 2007, 2008; Wolf et al., 2014). Similarly, EVs from *C. albicans* preferentially contain proteins associated with biofilm formation, cell wall remodeling, and stress adaptation (Vargas et al., 2015), whereas EVs from *P. brasiliensis* represent a defined subset of the extracellular proteome enriched in proteins involved in metabolism, signaling, and transport functions (Vallejo et al., 2011). Together, these findings support the concept that fungal EVs constitute specialized secretory compartments with selectively organized molecular cargo.

Environmental and physiological conditions further shape EV composition in a highly dynamic manner. In *C. albicans*, EV profiles differ markedly between planktonic and biofilm growth states, with biofilm-derived vesicles enriched in extracellular matrix components and antifungal resistance factors (Honorato et al., 2022; Zarnowski et al., 2018). Likewise, nutrient limitation and host-like growth conditions alter EV proteomes in *C. neoformans* (Marina et al., 2020), whereas oxidative stress and antifungal exposure modulate both EV abundance and molecular composition (Bleackley et al., 2019). In *H. capsulatum*, nutrient availability significantly influences EV-associated proteins, lipids, and metabolites, demonstrating that cargo loading is highly plastic and responsive to environmental cues (Cleare et al., 2020). Host-derived signals can also directly modulate EV biogenesis and composition. In *H. capsulatum*, antibody binding to fungal surface molecules altered EV size, enzymatic activity, and proteomic profiles, indicating that host–pathogen interactions dynamically influence vesicle-mediated secretion (Baltazar et al., 2016). At the population level, EV release is further influenced by quorum-sensing pathways and metabolic reprogramming under stress conditions, linking vesicle-mediated communication to coordinated fungal adaptation and community behavior (Bielska et al., 2018; Honorato et al., 2022; Nickerson et al., 2024).

Selective cargo loading extends beyond proteins to include nucleic acids. In *H. capsulatum*, EV-associated RNAs show little correlation with cellular transcript abundance and are instead enriched in transcripts related to stress responses, translation, and strain-specific small RNAs (Alves et al., 2019). In *C. neoformans*, deletion of *GRASP* yields EVs with a markedly altered RNA profile, particularly with accumulation of specific tRNA fragments and unique transcript sequences compared with wild-type cells (Peres da Silva et al., 2018). These findings provide strong evidence for regulated RNA sorting during EV biogenesis and support the idea that EV-associated RNAs contribute to communication with recipient cells.

Mechanistically, EV cargo loading is increasingly recognized as an active and energy-dependent process involving coordinated membrane remodeling, vesicle trafficking, and cargo recruitment pathways. ATP-dependent mechanisms regulate multiple stages of EV biogenesis, including cytoskeleton-mediated transport and ESCRT-dependent membrane scission (Henne et al., 2011). Molecular chaperones such as Hsp70 and Hsp90 may additionally contribute to selective cargo recruitment by stabilizing stress-responsive and virulence-associated proteins within vesicular pathways (Brandt et al., 2024). Consistent with this concept, network-based proteomic analyses revealed that EV-exclusive proteins in *C. haemulonii* cluster into functionally related pathways associated with proteasome assembly and fatty acid metabolism, supporting coordinated cargo packaging rather than stochastic incorporation (Oliveira et al., 2025).

Collectively, current evidence indicates that fungal EV biogenesis and cargo loading are highly dynamic and tightly regulated processes shaped by intracellular trafficking pathways, membrane organization, proteostasis networks, and environmental signals.

## Functional Roles of EVs in Fungal Pathogenesis

The recognition that fungal EVs serve as vehicles for intercellular communication has substantially advanced our understanding of fungal pathogenesis. Rather than functioning solely as cargo carriers, these membrane-bound structures contribute to complex host–pathogen interactions. Fungal EVs actively modulate host immune responses, deliver virulence determinants, and enhance survival under hostile conditions. This multifunctional nature positions EVs as key integrators of fungal physiology and pathogenesis, enabling opportunistic fungi to adapt to diverse host environments.

### Modulation of host immune responses

Fungal EVs interact extensively with host immune cells, including macrophages, dendritic cells, and epithelial cells, shaping both innate and adaptive immune responses. These interactions often produce a dual immunomodulatory effect that reflects a balance between immune activation and immune evasion (Fig. 2A).

Macrophages are primary cellular targets of fungal EV-mediated immune modulation. *C. neoformans* EVs exemplify this relationship by engaging with macrophages through receptor-mediated pathways that include Toll-like receptor 2 (TLR2) and TLR4 recognition of fungal pathogen-associated molecular patterns. Upon cellular uptake, these vesicles initiate signaling cascades that influence macrophage polarization toward M1- or M2-like phenotypes, ultimately affecting whether the immune response promotes pathogen clearance or tolerance (Colombo et al., 2020; Oliveira et al., 2010a). This immunological complexity is further reflected in EV-induced secretion of both pro- and anti-inflammatory mediators, including TNF-α, IL-10, TGF-β, and nitric oxide (NO), demonstrating their capacity to both stimulate and suppress immune responses (Oliveira et al., 2010a). This dual effect largely reflects EV cargo composition, particularly the presence of capsular components, which exert immunosuppressive and cytotoxic effects that promote fungal survival within host cells (Monari et al., 2005, 2006; Villena et al., 2008; Zaragoza et al., 2009). Notably, these vesicles can also traverse the blood-brain barrier (BBB) to enhance the pathogenesis of fungal brain infections, while modulating phagosome-lysosome fusion, creating protective intracellular sanctuaries for pathogen persistence (Fig. 2B) (Huang et al., 2012). Similarly, in *C. gattii*, EVs released by hypervirulent strains are rapidly internalized by macrophages and trafficked to cryptococcal phagosomes, where they enhance the intracellular proliferation of recipient fungal cells, demonstrating that EVs can directly manipulate the intracellular host environment to promote fungal survival (Bielska et al., 2018).

*C. albicans* EVs modulate immune responses via inducing dendritic cell and macrophage activation that results in coordinated production of IL-12, TNF-α, IL-10, and NO, along with upregulation of antigen presentation markers, including MHC-II and CD86 (Vargas et al., 2015). EVs also carry immunogenic molecules, including phosphatidylserine, glucosylceramide, and α-galactopyranosyl epitopes, which can trigger host immune responses (Noble et al., 2010; Peres da Silva et al., 2015a; Rittershaus et al., 2006; Wolf et al., 2015). Notably, immune activation is highly dependent on membrane composition, as EVs from phosphatidylserine-deficient *cho1*Δ mutants fail to activate NF-κB signaling, underscoring the importance of lipid architecture in immune recognition (Fig. 2B) (Wolf et al., 2015).

Across fungal species, EV-mediated immune modulation exhibits marked diversity. EVs from *M. sympodialis* induce adhesion molecule expression in keratinocytes and contribute to inflammatory response in skin (Vallhov et al., 2020), whereas EVs from *Aspergillus flavus* and *P. brasiliensis* promote M1 macrophage polarization and pro-inflammatory cytokine production (Brauer et al., 2020). In contrast, EVs from *H. capsulatum* suppress phagocytic activity and intracellular killing, illustrating their capacity to attenuate antifungal immunity (Baltazar et al., 2018). Interestingly, EVs from *S. brasiliensis* increase phagocytic activity while simultaneously enhancing fungal burden, suggesting that certain fungi exploit immune activation to favor infection (Fig. 2B) (Ikeda et al., 2018). EVs from *A. fumigatus* illustrate that immunomodulatory and protective outcomes do not always align: these EVs elicited only a partial pro-inflammatory response in macrophages, characterized by TNF-α induction and upregulated adhesion molecule and inducible nitric oxide synthase expression but no NETosis in neutrophils and minimal cytokine release from peripheral mononuclear cells. Nonetheless, prior exposure of *A. fumigatus* EVs significantly increased survival of *Galleria mellonella* larvae after fungal challenge. These observations indicate that EV-mediated protection can be achieved without a robust pro-inflammatory signature and that the magnitude of cytokine induction is not always predictive of in vivo protective efficacy (Freitas et al., 2023).

Beyond these species-specific examples, a broader question persists: can fungal EVs benefit the host? Accumulating evidence suggests that, under certain conditions, they can. *C. albicans* EVs have been shown to activate type I interferon signaling in host cells through the cGAS-STING pathway (Brown Harding et al., 2024), and the EV surface decoration with *O*- and *N*-linked mannans, β-1,3-glucans, and chitin oligomers engaged TLR4 and dectin-1 to drive a protective TLR4-dependent immune response (Honorato et al., 2024; Rizzo et al., 2021). Notably, *C. albicans* EVs reduced the severity of experimental keratitis, lowered fungal burden, and improved disease outcome when mice were pre-exposed to them (Duan et al., 2024), consistent with earlier reports that fungal EVs can prime macrophage antifungal activity and induce protective responses (Almeida et al., 2017; Hatanaka et al., 2019; Oliveira et al., 2010a, 2023; Peres da Silva et al., 2015a; Rizzo et al., 2021; Vargas et al., 2015).

The opposite can also be true. EVs from *C. albicans*, *C. neoformans*, and *A. fumigatus* are internalized by macrophages at different rates and elicit distinct cytokine and signaling responses; notably, cell wall components on *C. neoformans* and *A. fumigatus* EVs can impair macrophage uptake and dampen innate immune activation (Kwaku et al., 2025). In *P. brasiliensis*, pretreatment of mice with EVs before infection with a virulent isolate worsened disease severity, increasing the production of pulmonary TNF-α, IFN-γ, IL-6, and MCP-1 (Octaviano et al., 2022), and *S. brasiliensis* EVs were similarly associated with increased fungal burden in experimental sporotrichosis (Ikeda et al., 2018). In line with these findings, *C. neoformans* mutants with reduced EV production and attenuated virulence regained their pathogenicity when supplemented with EVs from wild-type cells (Castelli et al., 2024). This question stands in contrast to the previous discussion on the immunoprotective effects of fungal EVs, highlighting their potential to either enhance or suppress antifungal immune responses. These seemingly contradictory effects likely depend on the fungal species, EV composition, and experimental conditions used to stimulate host cells. However, one undeniable observation remains: fungal EVs can have paradoxical effects, underscoring the need for further immunological investigation.

Although less extensively studied, EVs from dermatophytes and other environmental fungi also exhibit biologically relevant functions. *Trichophyton interdigitale* produces EVs capable of modulating macrophage and keratinocyte responses, suggesting roles in immune evasion and tissue colonization (Bitencourt et al., 2018). Mechanistically, *T. interdigitale* EVs induce TLR2-dependent release of TNF-α, IL-6, and IL-1β by bone marrow-derived macrophages, drive polarization toward the classical M1 phenotype, and enhance both phagocytosis and fungicidal killing of conidia, indicating that dermatophyte EVs can prime an effective antifungal innate response despite the comparatively mild clinical presentation of dermatophytosis (Bitencourt et al., 2018). EVs from the chromoblastomycosis agents *Fonsecaea pedrosoi* and *F. nubica* further illustrate growth-condition-dependent immunomodulation: EVs isolated from nutrient-rich medium, but not from minimal medium, induced robust TNF, IL-1β, IL-10, and nitric oxide production by macrophages, with IL-1β release dependent on nigericin-mediated inflammasome priming, consistent with these EVs acting as a first signal for inflammasome activation (Las-Casas et al., 2022). The neurotropic black yeast *Exophiala dermatitidis* produces EVs with unique cytotoxic properties, with melanin-containing vesicles showing significantly greater neurotoxicity than non-melanized EVs, highlighting a potential role in host tissue damage (Lavrin et al., 2020). Collectively, these findings indicate that fungal EVs act as finely regulated immunomodulatory mediators capable of either enhancing host defense or facilitating immune evasion, depending on fungal species, EV composition, and host context.

### Delivery of virulence determinants

Fungal EVs serve as efficient delivery systems for virulence-associated molecules, enabling their protected transport and release within host environments. Encapsulation within lipid bilayers shields cargo from extracellular degradation while preserving biological activity. In *C. neoformans*, EVs transport key virulence factors, including GXM, laccase, and urease (Fig. 2B). GXM, the principal capsular polysaccharide, is essential for immune evasion, inhibiting macrophage-mediated killing and modulating host responses (Monari et al., 2005, 2006; Villena et al., 2008; Zaragoza et al., 2009). EV-mediated export of GXM is essential for capsule assembly and pathogenicity, providing a mechanism for polysaccharide translocation across the thick fungal cell wall. Similarly, EV-associated laccase contributes to melanin production, enhancing resistance to oxidative stress and phagocytosis while maintaining enzymatic activity through vesicle-mediated protection (Coelho et al., 2014; Eisenman et al., 2009). These vesicles also facilitate fungal dissemination by promoting transmigration across the BBB, leading to cryptococcal meningoencephalitis (Huang et al., 2012). In *C. gattii*, EVs derived from highly virulent strains enhance intracellular proliferation of less virulent isolates through a “division of labor” mechanism, illustrating EV-mediated propagation of virulence traits (Bielska et al., 2018).

EV cargo also includes hydrolytic enzymes, such as proteases and phospholipases, that contribute to tissue invasion and immune evasion (Gil-Bona et al., 2015). In *C. albicans*, cargo composition is directly influenced by lipid biosynthetic pathways, with EVs from wild-type *C. albicans* enriched in virulence-associated proteins including the phospholipase Plb3, the adhesin Sim1, and the proteinase Prd1, whereas mutants deficient in these pathways exhibit altered cargo profiles and reduced pathogenicity (Fig. 2B) (Wolf et al., 2015). In addition, EVs from *Candida* species other than *C. albicans* contain multifunctional, or “moonlighting,” proteins involved in adhesion, immune modulation, and virulence (Karkowska-Kuleta et al., 2020).

EV-associated small RNAs have also emerged as important mediators of host–pathogen interactions. Fungal EVs can deliver small RNAs to recipient cells, where they may modulate host gene expression through RNAi-related mechanisms (Peres da Silva et al., 2015b). Similarly, EVs from *H. capsulatum* contain RNA-binding proteins associated with RNAi machinery, suggesting that vesicles may transport both regulatory RNAs and components required for gene silencing (Alves et al., 2019). Similarly, EVs from *M. sympodialis* carry small RNAs capable of inducing inflammatory responses in human keratinocytes, including upregulation of adhesion molecules and cytokine signaling pathways, highlighting a role for EV-mediated RNA delivery in immune modulation (Fig. 2B) (Johansson et al., 2018; Vallhov et al., 2020).

### Roles in cell-cell communication and antifungal resistance

Beyond their direct pathogenic effects, fungal EVs contribute to fungal fitness by promoting biofilm formation, stress adaptation, and antifungal resistance (Fig. 2A). Biofilms are structured microbial communities embedded in an extracellular matrix that protects against environmental stress and antimicrobial agents (Kowalski et al., 2020). In *C. albicans*, EVs are integral to biofilm development. Biofilm-derived EVs are enriched in proteins and polysaccharides, including mannan and glucan, which resemble matrix components and contribute to matrix assembly and stability (Mitchell et al., 2015; Zarnowski et al., 2018, 2021). Disruption of EV production, as observed in ESCRT mutants, reduces matrix formation and increases susceptibility to antifungal agents, whereas supplementation with wild-type EVs restores matrix integrity and drug resistance (Zarnowski et al., 2018, 2021).

In addition, EVs facilitate sophisticated cell-to-cell communication within biofilm communities by transporting signaling molecules, including farnesol and tyrosol, that regulate development and morphological transitions. This vesicle-mediated signaling enables coordinated population responses that enhance community survival. Biofilm-associated EVs confer greater antifungal resistance than those derived from planktonic forms, suggesting stress-induced cargo modifications that promote resistance (Zarnowski et al., 2018). EV production increases under stress conditions, with vesicles carrying antioxidant enzymes and protective factors that enhance community-level resilience (Albuquerque et al., 2008; Wolf et al., 2015).

EV-mediated communication is not restricted to cells of the same species, and recent work shows that the outcome of interspecies EV exchange ranges from cooperation to antagonism depending on the biofilm life-cycle stage and species pairing. During biofilm initiation, EVs from *Candida* species restored adhesion defects only within the same species, while cross-species exchange typically reduced adhesion below baseline, indicating interference competition during early colonization (Zarnowski et al., 2022). This contrasts with the cooperative behavior reported for mature biofilm-phase EVs, suggesting that interspecies signaling shifts from competition to cooperation as the biofilm develops. Supporting this, exogenous EVs from *C. albicans* and *C. auris* enhanced each other's biofilm adhesion and dispersion, and EVs from wild-type *Cryptococcus* species partially restored virulence-associated gene expression (*CAP59*, *LAC1*, *URE1*) and reduced antifungal susceptibility in an acapsular *C. neoformans* mutant, even across genus boundaries, although the full capsule phenotype was not reconstituted. Cross-species EV transfer also disseminated adaptive drug-resistance signals, as *C. auris*-derived EVs induced *ERG11* upregulation and reduced azole susceptibility in recipient *C. albicans* cells (Piraine et al., 2026). Together, these findings indicate that EV-mediated interspecies communication is shaped by biofilm stage, species identity, and the specific cargo exchanged, rather than following a single cooperative or antagonistic rule.

EVs also contribute to antifungal resistance through the transport of drug efflux pumps and stress-response proteins (Fig. 2A). The presence of efflux pumps such as Cdr1 and Mdr1 suggests that EVs may facilitate the dissemination of resistance traits within fungal populations (Dawson et al., 2020; Holmes et al., 2008; Luna-Tapia et al., 2015). In addition, EVs enriched in heat shock proteins and oxidative stress factors enhance tolerance to antifungal and environmental stress. Species-specific adaptations are particularly evident in emerging multidrug-resistant pathogens. *C. auris* produces EVs with a protein cargo profile markedly distinct from that of *C. albicans*, including enrichment of adhesion-associated proteins and neutral lipids that correlate with enhanced epithelial interaction and pathogenic potential (Zamith-Miranda et al., 2021a). EV composition in *C. auris* is also dynamically remodeled under antifungal stress, as exposure to caspofungin alters both vesicle production and cargo composition, supporting a role for EVs in adaptive stress responses and antifungal resistance mechanisms (Amatuzzi et al., 2022).

Overall, fungal EVs represent highly versatile and tightly regulated systems that integrate immune modulation, virulence factor delivery, and environmental adaptation. Their multifunctional nature underscores their central role in fungal pathogenesis, reflecting both conserved mechanisms and species-specific adaptations that enable successful colonization of diverse host niches (Fig. 2B).

### EV stability and fate in host and in vitro environments

Although fungal EVs exhibit diverse biological functions, their pathogenic and therapeutic relevance ultimately depends on their structural integrity and biostability under physiological conditions.

Some host-derived factors are reported to influence EV stability and extracellular persistence. Serum albumin, the most abundant plasma protein, rapidly disrupts cryptococcal EVs, likely through sequestration of membrane-associated lipids such as ergosterol and fatty acids (Wolf et al., 2012). Albumin-mediated vesicle destabilization significantly reduces EV uptake by macrophages, suggesting that fungal EVs not promptly internalized in vivo may undergo rapid extracellular degradation and premature cargo release. Host lectins can similarly interfere with fungal EV integrity. Galectin-3 (Gal-3), a β-galactoside-binding lectin induced during cryptococcal infection, exerts direct lytic activity against *C. neoformans* EVs while simultaneously inhibiting fungal growth (Almeida et al., 2017). Comparable Gal-3-dependent disruption has also been reported for *P. brasiliensis* EVs, indicating that lectin-mediated vesicle destabilization may represent a conserved host defense mechanism targeting fungal EV-mediated communication (Hatanaka et al., 2019).

The susceptibility of fungal EVs to host-mediated disruption has important implications for both pathogenesis and therapeutic development. Premature vesicle lysis may restrict long-distance fungal communication while simultaneously exposing immunogenic cargo to host immune surveillance. Conversely, factors that enhance EV persistence could promote sustained delivery of virulence-associated molecules during infection. Improved understanding of the molecular determinants governing EV stability, cargo retention, and extracellular persistence will therefore be essential for clarifying the biological relevance of fungal EVs in vivo and advancing their potential application as diagnostic biomarkers, vaccine platforms, and therapeutic delivery systems.

EV stability in vitro matters as it can directly influence their translational use in the future. Current evidence suggests that fungal EVs generally retain structural and functional integrity under short-term storage conditions, although stability appears to vary among species. Studies in *C. albicans* have demonstrated that EVs preserve their morphology and immunostimulatory activity following storage under multiple temperature conditions, whereas *C. neoformans* EVs remain stable in culture medium for at least 72 h (Vargas et al., 2020; Wolf et al., 2012). Nevertheless, insights from mammalian EV systems indicate that freeze–thaw stress and long-term storage can alter vesicle integrity, cargo stability, and uptake efficiency, highlighting the need for standardized preservation strategies specifically optimized for fungal EVs (Kusuma et al., 2018; Maroto et al., 2017; Wu et al., 2021).

## Applications of Fungal Extracellular Vesicles

The global burden of invasive fungal infections continues to rise, driven by the emergence of new pathogenic species, the spread of antifungal resistance, and the growing population of immunocompromised individuals due to increased use of immunosuppressive chemotherapeutics. Despite this escalating global challenge, therapeutic options for invasive mycoses remain limited, and no antifungal vaccine has yet been licensed for clinical use (Denning, 2024; Fisher et al., 2018; Nami et al., 2019; Nicola et al., 2019). The expanding understanding of fungal EV biology has revealed new opportunities for translational application. Their intrinsic immunogenicity, structural stability, and species-specific cargo composition position them as attractive candidates for diagnostic development, therapeutic targeting, vaccine design, and molecular delivery systems (Fig. 3) (Freitas et al., 2019; Honorato et al., 2021).

### Fungal EVs as diagnostic biomarkers

Current diagnostic approaches for invasive fungal infections, including culture, histopathology, serological assays, and molecular detection, frequently lack the sensitivity, specificity, or rapid turnaround required for early and accurate diagnosis (Fisher et al., 2018; Mendonça et al., 2022). In this context, fungal EVs have emerged as attractive biomarker candidates because they carry stable and disease-relevant molecular cargo that reflects fungal physiology, pathogenic state, and host adaptation.

EVs from several pathogenic fungi are recognized by patient sera and carry highly immunogenic molecules associated with fungal virulence (Fig. 3A). In *C. neoformans*, EVs are enriched in GXM, mannoproteins, melanin-associated components, and immunogenic antigens such as Mp88 and Cda1, all of which are closely linked to cryptococcal dissemination and pathogenicity (Mota et al., 2025; Rizzo et al., 2021; Rodrigues et al., 2008; Wolf et al., 2014). Similarly, EVs from *M. sympodialis* contain the allergens Mala s 1 and Mala s 7, suggesting their potential utility as biomarkers of inflammatory skin disorders and atopic dermatitis (Johansson et al., 2018; Vallhov et al., 2020). Comparative proteomic analyses have additionally identified conserved EV-associated proteins across *H. capsulatum*, *P. brasiliensis*, and *C. neoformans*, supporting the existence of shared fungal EV marker candidates (Albuquerque et al., 2008; Mota et al., 2025; Vallejo et al., 2011).

EV-associated RNAs represent another promising biomarker class. Fungal EVs package miRNA-like molecules, tRNA fragments, and RNAs associated with spliceosome assembly and stress adaptation, many of which differ substantially from intracellular RNA populations (Munhoz da Rocha et al., 2021; Peres da Silva et al., 2015b). Because EV-associated RNAs are stable and species-specific, they may provide useful indicators of fungal adaptation and infection status.

### EV-based vaccines and immunotherapeutic platforms

The strong immunogenicity of fungal EVs, packed with fungal antigens, glycans, lipids, and immunomodulatory molecules, has generated substantial interest in their application as vaccine and immunotherapeutic platforms (Fig. 3B). Their non-replicative nature and enrichment in pathogen-associated molecular patterns further enhance their attractiveness as vaccine candidates (Bachmann and Jennings, 2010).

Fungal EVs stimulate both innate and adaptive immune responses. *C. albicans* EVs activate dendritic cells and macrophages, promoting cytokine production and upregulation of MHC-II and CD86 (Vargas et al., 2015), whereas EVs from *Talaromyces marneffei* induce inflammatory signaling associated with downstream T-cell activation (Yang et al., 2021). Proteomic analyses have revealed enrichment of major fungal antigens within EV populations. *C. albicans* EVs contain the immunodominant antigens Mp65, Bgl2, and Eno1 (Vargas et al., 2015), whereas *M. sympodialis* EVs are enriched in allergens (Johansson et al., 2018). In *P. brasiliensis*, EVs carry the glycoprotein gp43 and its immunogenic peptide P10, which induces protective Th1-mediated immunity in murine infection models (Vallejo et al., 2012). Similarly, cryptococcal EVs are enriched in mannoproteins, including Mp88, Mp98, and Cda1, all of which were previously identified as protective antigens (Huang et al., 2002; Levitz et al., 2001; Mota et al., 2025; Rizzo et al., 2021).

Protective effects of fungal EV-based immunization have now been demonstrated in multiple experimental models. Pretreatment with EVs from *C. albicans*, *C. neoformans*, or *A. flavus* improves survival in *G. mellonella* infection models (Brauer et al., 2020; Colombo et al., 2020; Vargas et al., 2015). In murine candidiasis, repeated administration of *C. albicans* EVs induces antifungal IgG responses, reduces fungal burden, and confers complete protection against lethal infection (Vargas et al., 2020). Similarly, EV immunization prolongs survival in murine models of cryptococcosis and pulmonary aspergillosis (Rizzo et al., 2021; Souza et al., 2019). Notably, mice immunized with EVs derived from acapsular cryptococcal mutants exhibit greater protection following challenge with virulent strains, indicating that EV surface composition critically influences vaccine efficacy (Rizzo et al., 2021). The cytokine profile elicited by EV immunization can depend on adjuvant use: in murine candidiasis, mice vaccinated with *C. albicans* EVs plus adjuvant showed elevated IL-12p70, TNF-α, and IFN-γ, whereas adjuvant-free immunization instead increased IL-12p70, TGF-β, IL-4, and IL-10, indicating that EVs can shift the host response toward distinct cytokine profiles depending on co-administered immunostimulants (Vargas et al., 2020). Vaccine potential is not restricted to pathogenic fungi: EVs from the non-pathogenic yeast *Saccharomyces cerevisiae* carry β-D-glucan and heat shock protein 70 (Hsp70) and are internalized by macrophage-like and dendritic cells, stimulating TNF-α and IL-6 production and upregulating the maturation markers CD40, CD80, and CD86 through a TLR2-dependent mechanism (Higuchi et al., 2023). This finding raises the possibility that engineered or heterologous fungal EV platforms could be developed as antigen-delivery vehicles independent of pathogen virulence.

### Therapeutic targeting of fungal EV pathways

Because fungal EVs contribute directly to virulence factor export, immune modulation, biofilm formation, and antifungal resistance, interference with EV biogenesis and cargo trafficking has emerged as a potential antifungal strategy (Fig. 3C). Fungal EV production depends on interconnected pathways that include ESCRT-dependent endosomal sorting, GRASP-mediated, PM-budding and autophagy-associated unconventional secretion pathways (Oliveira et al., 2010b; Panepinto et al., 2009). Disruption of these pathways markedly alters EV production and fungal pathogenicity in *C. neoformans* and *C. albicans* (Oliveira et al., 2010b; Panepinto et al., 2009; Zarnowski et al., 2018).

EV-mediated communication also contributes to community-wide adaptation and virulence dissemination. In *C. albicans*, biofilm-derived EVs transport matrix polysaccharides, stress-response proteins, and drug-efflux transporters such as Cdr1 and Mdr1, thereby promoting antifungal resistance within fungal populations (Dawson et al., 2020). Restoration of wild-type EVs rescues matrix integrity and antifungal resistance, demonstrating that EV-mediated cargo delivery is functionally required for biofilm-associated persistence. A complementary example was described in *C. gattii*, where EVs released by hypervirulent strains are internalized by infected macrophages and trafficked to cryptococcal phagosomes, triggering rapid intracellular proliferation of neighboring less virulent strains through a species-specific “division of labour” mechanism (Bielska et al., 2018). Because this phenomenon depends on intact vesicles carrying functional protein and RNA cargo, it highlights EV-mediated communication as a potential therapeutic target. However, the identification of fungal-specific components involved in EV biogenesis and cargo loading is a prerequisite for the development of EV-based therapeutic strategies, as it would enable selective targeting of fungal EV pathways while minimizing off-target effects on host cells.

The relationship between EV production and antifungal resistance is species- and context-dependent, and no single unifying pattern has yet emerged. In *C. neoformans*, transcription-factor mutants with markedly reduced EV output show altered fluconazole susceptibility, and reduced EV production is generally associated with increased drug resistance (Rizzo et al., 2023). In contrast, in *C. auris*, exogenously added EVs increase amphotericin B resistance in a dose-dependent manner without affecting *C. albicans*, indicating that EVs can actively disseminate resistance rather than simply correlating with it (Chan et al., 2022). The relationship reverses again in biofilms: ESCRT-defective *C. albicans* mutants produce fewer EVs, accumulate less matrix polysaccharide, and become hypersusceptible to fluconazole, while exogenous wild-type EVs restore both matrix architecture and drug resistance (Zarnowski et al., 2018, 2021). Comparable EV-dependent regulation of biofilm cargo and resistance phenotypes has since been confirmed across *C. tropicalis*, *C. parapsilosis*, *C. glabrata*, and *C. auris*, with cross-species EV complementation able to restore biofilm phenotypes (Zarnowski et al., 2022). Together, these findings show that reduced EV output can be associated with either increased resistance (planktonic *C. neoformans*) or increased susceptibility (*C. albicans* biofilms), while exogenous EV exposure can independently confer resistance (*C. auris*), underscoring that EV-mediated drug resistance operates through distinct, organism- and context-specific mechanisms rather than a single conserved pathway.

### EVs as drug delivery vehicles and other biotechnology tools

The intrinsic capacity of fungal EVs to transport bioactive molecules has generated increasing interest in their potential application as nanoscale delivery systems and other biotechnology platforms (Fig. 3D). Their lipid bilayer protects cargo from extracellular degradation while facilitating interaction with target cells, and their reported ability to traverse biological barriers, including the BBB, further highlights their translational potential as therapeutic delivery vehicles (Huang et al., 2012; van Niel et al., 2018). RNA-based therapeutic approaches represent another promising area of fungal EV biotechnology. Experimental approaches involving vesicle-protected RNAs and host-induced gene silencing have already demonstrated potential for antifungal intervention and pathogen control (Cai et al., 2018; Rodrigues et al., 2015).

### Limitations in translational use of fungal EVs

Major limitations of fungal EV applications include low vesicle yield, incomplete understanding of cargo-loading and targeting mechanisms, heterogeneity of vesicle populations, and the lack of standardized isolation and production protocols (Rizzo et al., 2020; Rodrigues et al., 2016). A practical barrier specific to EV-based vaccine development is the scaled-up and reproducible production of EVs at quantities suitable for preclinical and clinical testing; recent methodological advances in fungal EV isolation and analysis may help address this bottleneck (Reis et al., 2019; Rizzo et al., 2023). Moreover, the absence of universally accepted fungal EV markers, analogous to the mammalian tetraspanins CD9, CD63, and CD81, hinders standardized EV purification, characterization, and cross-study comparison (Bleackley et al., 2019; Dawson et al., 2020). These technical challenges are further compounded by incomplete understanding of the biological consequences of EV-mediated cargo delivery. Fungal EVs can exert paradoxical effects depending on species, cargo composition, and host context: although several EV preparations confer protection in infection models, EVs from *S. brasiliensis* increase fungal burden and lesion severity in murine sporotrichosis (Ikeda et al., 2018), *P. brasiliensis* EVs can worsen disease severity when administered before infection, with increased production of pulmonary TNF-α, IFN-γ, IL-6, and MCP-1 (Octaviano et al., 2022), and *C. neoformans* mutants with reduced EV production and attenuated virulence can regain pathogenicity when supplemented with wild-type vesicles (Castelli et al., 2024). This duality represents a substantive safety consideration for EV-based vaccines or therapeutic candidates. A related but underexplored risk is that EVs can act across species boundaries, transferring drug-resistance signals or virulence-associated cargo between phylogenetically divergent fungi (Piraine et al., 2026); the consequences of administering an EV-based product in the context of polymicrobial colonization, common in clinical settings, remain unknown.

Importantly, nearly all the evidence supporting diagnostic, vaccine, and therapeutic applications of fungal EVs to date derives from in vitro assays and animal infection models; validation in patient-derived samples remains exceedingly rare across all application categories (Table 2). Bridging this gap will require not only scalable and standardized EV production but also dedicated clinical validation studies, established reference materials, consortium-based standardization efforts analogous to MISEV for mammalian EVs, and engineered or non-pathogenic fungal EV chassis that could decouple antigen delivery from the safety concerns associated with pathogen-derived vesicles. These observations underscore that comprehensive cargo characterization, immunological safety profiling, and careful EV engineering are prerequisites for translational application.

## Challenges and Perspectives

Nearly two decades after the first formal description of fungal EVs in *C. neoformans* (Rodrigues et al., 2007), the field has progressed from establishing that fungi release membrane-bound vesicles to dissecting the molecular mechanisms governing their biogenesis, cargo composition, and biological functions. These vesicles are no longer viewed as passive byproducts of secretion, but rather as multifunctional regulators that integrate unconventional protein export, stress adaptation, biofilm assembly, host immune modulation, and trans-cell-wall delivery of virulence determinants (Bielska et al., 2018; Oliveira et al., 2010a, 2010b; Rodrigues et al., 2008; Vargas et al., 2015). This conceptual shift has positioned fungal EVs at the intersection of fungal cell biology and pathogenesis, with direct implications for diagnostics, vaccine development, and antifungal therapeutics.

Despite these advances, several fundamental mechanistic questions remain unresolved. Foremost among them is how membrane-bound vesicles traverse the dense, polysaccharide-rich fungal cell wall, a barrier once considered incompatible with vesicular export. Direct visualization of EV transit through intact wall structures remains technically challenging, and the relative contributions of individual mechanisms likely vary among fungal species and environmental conditions. Future studies combining high-resolution live-cell imaging, cryo-EM, and targeted genetic perturbation of cell wall biosynthesis and remodeling pathways will be essential to resolve these questions. In parallel, comparative analyses across diverse fungal lineages will help clarify how differences in cell wall composition, thickness, and architecture influence EV release and extracellular trafficking.

Equally important are unresolved questions regarding the molecular pathways governing EV biogenesis and cargo selection. Although ESCRT-dependent endosomal sorting and GRASP-mediated unconventional secretory routes have been implicated in fungal EV formation, the molecular machinery driving membrane curvature, vesicle scission, and cargo loading in fungal EVs remains poorly characterized. Emerging evidence links intracellular proteostasis and glycosylation fidelity to EV cargo regulation. Disruption of the *N*-glycan-dependent ERQC system in *C. neoformans* significantly alters EV abundance, size distribution, and protein composition while impairing the secretion of major virulence determinants (Mota et al., 2025). In parallel, recent studies demonstrated that *O*-mannosylation critically regulates the stability, secretion, and immune recognition of the EV-associated mannoproteins Mp88 and Cda1 (Thak et al., 2025), indicating that glycan architecture may directly shape EV immunogenicity and host interactions.

Another critical yet underexplored challenge concerns the stability and fate of fungal EVs within host environments. The extent to which EVs remain structurally intact during infection likely has implications for long-distance communication, immune recognition, and therapeutic application. Premature vesicle disruption may limit intercellular signaling while simultaneously exposing immunogenic cargo to host surveillance systems. Although albumin-mediated destabilization has been described for *C. neoformans* EVs (Wolf et al., 2012), comparable studies across other pathogenic fungi remain scarce. Whether EVs produced during active infection exhibit enhanced resistance to host-mediated disruption compared with laboratory-derived vesicles also remains unknown. Addressing these questions will be critical for understanding the true contribution of EV-mediated communication during infection and for guiding the development of EV-based therapeutic platforms.

Current evidence increasingly supports the view that fungal EVs function as coordinated regulators of population-level adaptation and pathogenicity. By coupling secretion, stress adaptation, and intercellular communication, EVs enable fungal populations to disseminate virulence determinants, modulate host immunity, and potentially spread adaptive traits such as antifungal resistance (Bielska et al., 2018; Chan et al., 2022; Zarnowski et al., 2018, 2021). Understanding how these processes are coordinated at the molecular and community levels will be essential for defining the broader contribution of EVs to fungal pathogenesis. Further integration of multi-omics approaches, live-cell imaging, and targeted genetic perturbation studies will be essential to elucidate the molecular principles governing EV biogenesis and cargo selection and to define how EV-mediated secretion contributes to fungal adaptation, intercellular communication, and pathogenicity.

The translational potential of fungal EVs is becoming increasingly evident, although substantial technical and biological challenges remain before clinical implementation becomes feasible. As diagnostic biomarkers, EVs offer the possibility of detecting infection-specific molecular signatures in patient-derived fluids with greater sensitivity and species discrimination than currently available antigen-based assays. As vaccine platforms, fungal EVs combine native multivalent antigen presentation with intrinsic immunostimulatory properties, an especially attractive feature given the absence of licensed antifungal vaccines (Rizzo et al., 2021; Vargas et al., 2020).

Progress in multi-omics profiling, scalable purification strategies, and EV engineering will therefore be essential for advancing fungal EV-based diagnostics, therapeutics, and biotechnology applications. Advances in mammalian EV engineering, including cargo optimization and surface ligand modification, provide a valuable conceptual framework for future fungal EV manipulation (Alvarez-Erviti et al., 2011; Wiklander et al., 2019).

Collectively, fungal EVs are emerging as central regulators of fungal biology and promising tools for combating the growing global burden of fungal disease. Bridging mechanistic details with translational application will require sustained collaboration among fungal cell biologists, clinical mycologists, and biotechnology researchers, particularly in the context of the WHO fungal priority pathogens list (Fisher and Denning, 2023), which underscores the urgent need for new antifungal strategies.

## Figures and Tables

**Fig. 1.**
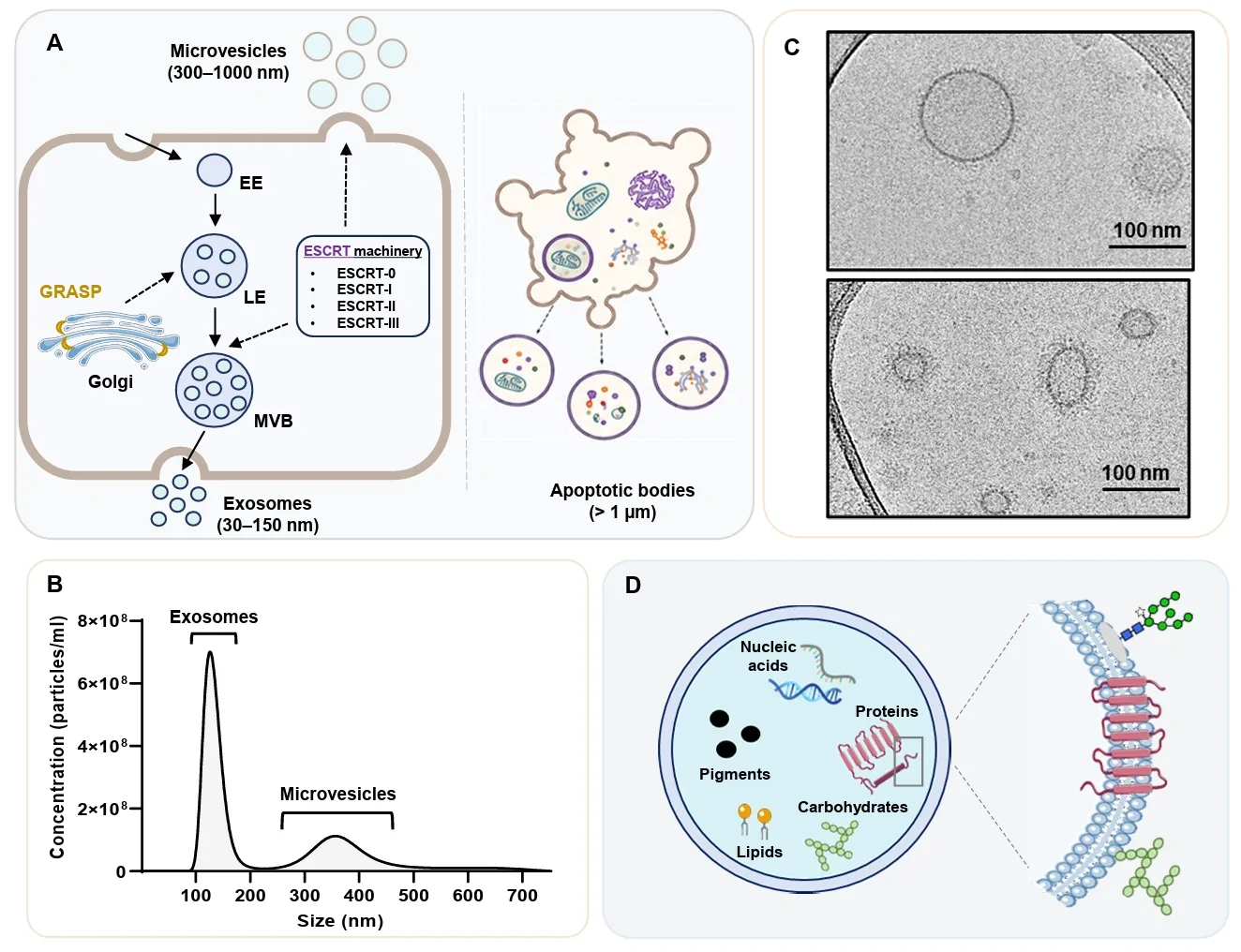
Biogenesis pathways and molecular cargo composition of fungal extracellular vesicles (EVs). (A) Biogenesis pathways of EVs. Fungal EVs originate through multiple intracellular and plasma membrane (PM)–associated biogenetic pathways. Exosome-like EVs are generated through the endosomal pathway, in which early endosomes (EEs) mature into late endosomes (LEs) and multivesicular bodies (MVBs). Intraluminal vesicles (ILVs) are formed within MVBs through membrane invagination and scission mediated by the endosomal sorting complex required for transport machinery, including ESCRT-0, ESCRT-I, ESCRT-II, and ESCRT-III. Fusion of MVBs with the PM releases ILVs extracellularly as exosome-like EVs. Golgi reassembly and stacking protein (GRASP)-associated unconventional secretion pathways also contribute to EV biogenesis and cargo trafficking through Golgi-associated secretory mechanisms. In contrast, larger microvesicle-like EVs are generated by direct outward budding of the PM. Additional extracellular vesicular structures, including apoptotic bodies, are released during programmed cell death and cellular fragmentation and may contain organelle fragments, nucleic acids, and cytoplasmic components derived from disassembling cells. (B) Representative nanoparticle tracking analysis (NTA) profile showing the heterogeneous size distribution of *C. neoformans* EV populations. (C) Representative cryogenic electron microscopy (cryo-EM) images illustrating the morphological heterogeneity of *C. neoformans* EVs. Panels (B) and (C) were adapted from Mota et al. (2025). (D) Schematic summary of major molecular cargo packaged within fungal EVs. The heterogeneous composition of fungal EVs reflects regulated cargo loading processes associated with fungal physiology, stress adaptation, and pathogenicity.

**Fig. 2.**
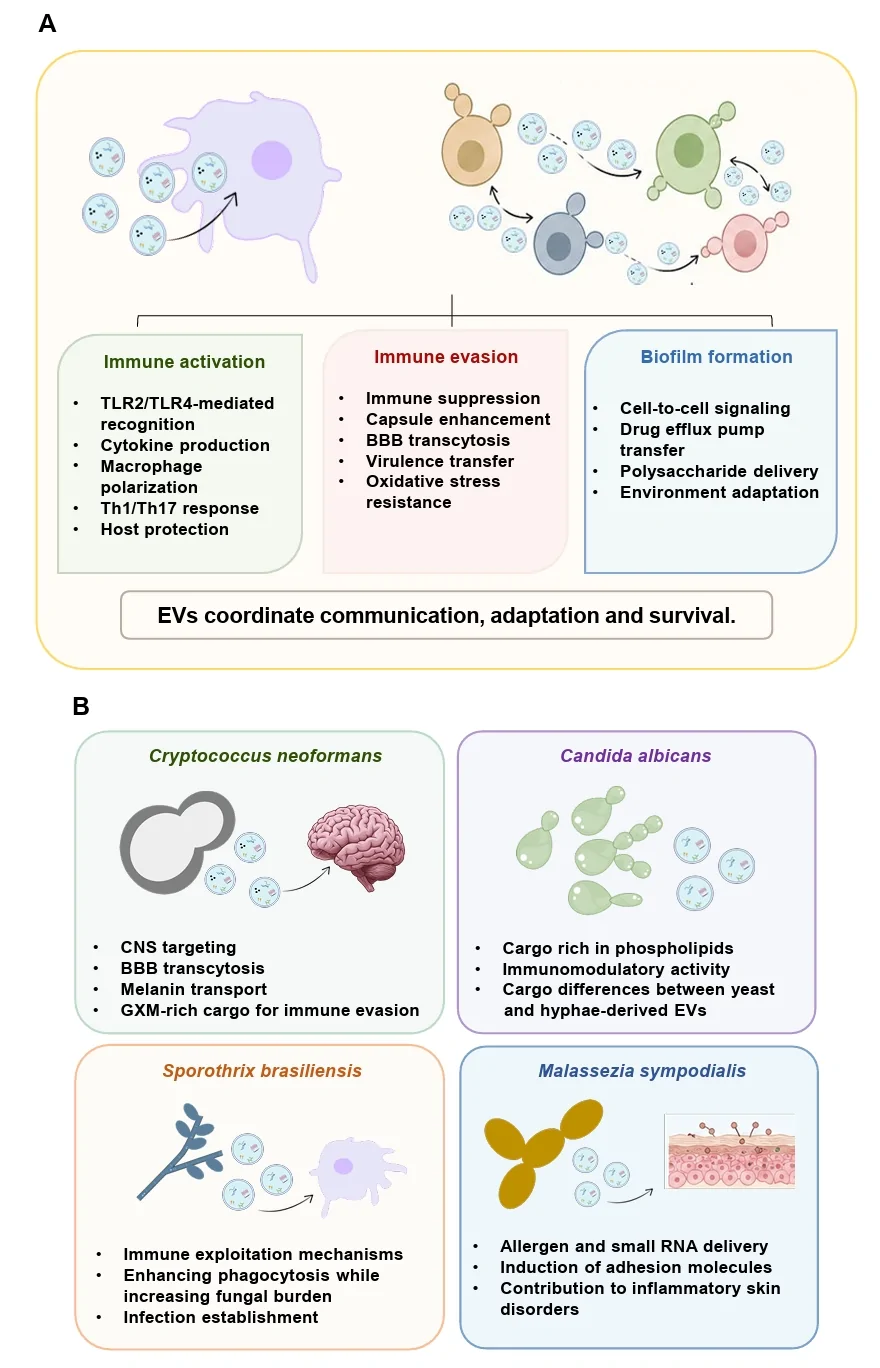
Host interaction, cell–cell communication, and species-specific adaptations mediated by fungal EVs. (A) EVs in cell–cell interactions. Fungal EVs mediate bidirectional communication between fungal cells and host immune cells, promoting diverse outcomes, including immune activation, immune evasion, biofilm formation, and environmental adaptation. During host–pathogen interactions, EVs induce cytokine production, macrophage polarization, and Th1/Th17 responses, while also contributing to immune suppression, oxidative stress resistance, virulence factor transfer, and blood–brain barrier (BBB) transcytosis in host cells. EV-mediated fungal cell–cell communication additionally supports stress adaptation, dissemination of antifungal resistance, biofilm coordination, and community-level signaling. Collectively, these functions position EVs as central regulators of fungal communication, adaptation, and survival. (B) Species-specific EV adaptation to host environments. Comparative analysis highlights species-specific EV adaptations associated with distinct pathogenic strategies and host niches. *C. neoformans* EVs are associated with central nervous system (CNS) targeting, BBB traversal, melanization, and GXM-mediated immune evasion. *C. albicans* EVs are enriched in phospholipid-associated cargo and exhibit distinct cargo profiles between yeast- and hypha-derived vesicles. *S. brasiliensis* EVs promote immune exploitation mechanisms that enhance phagocytosis while increasing fungal burden. *M. sympodialis* EVs deliver allergens and small RNAs that induce adhesion molecule expression and contribute to inflammatory skin responses.

**Fig. 3.**
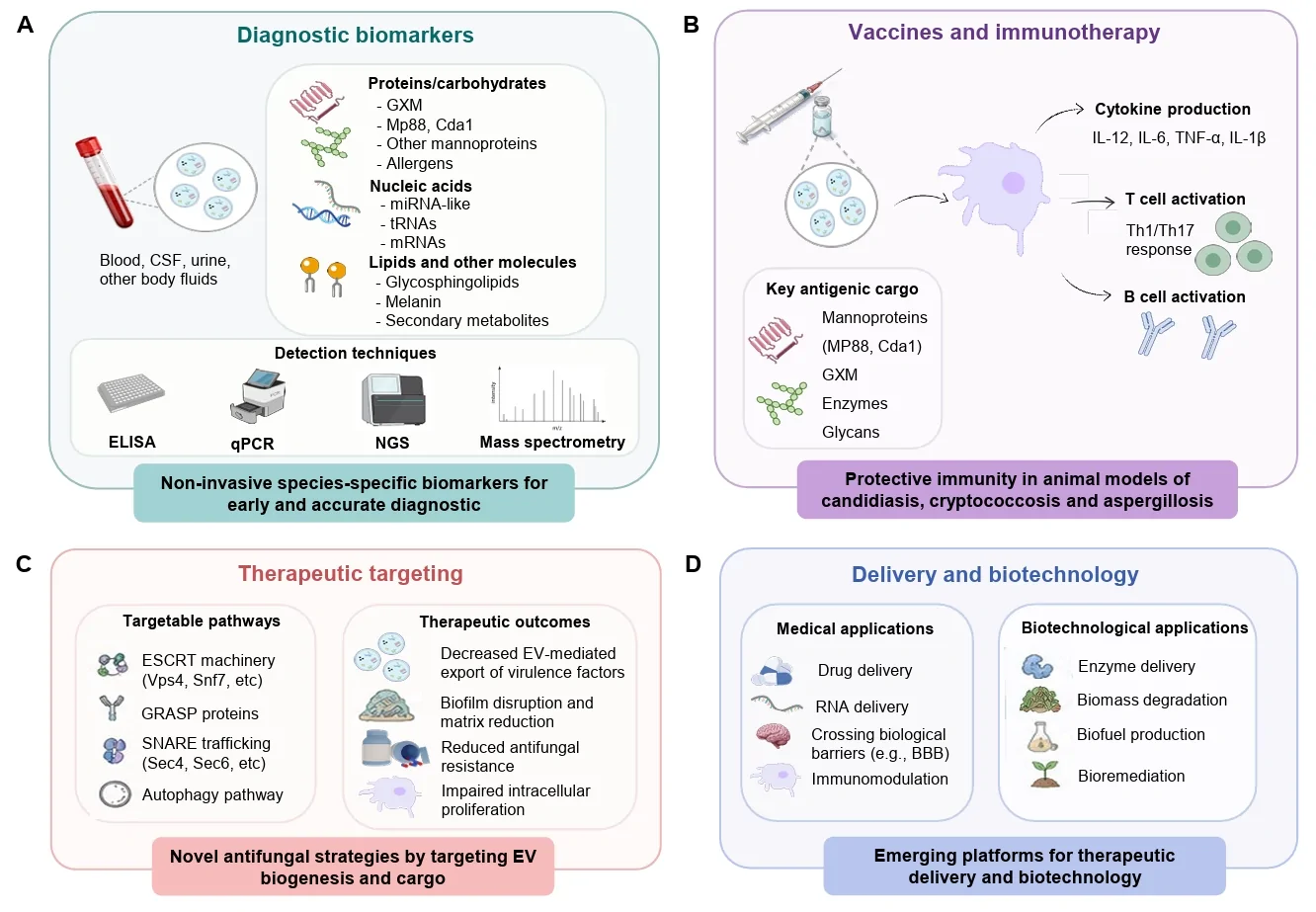
Translational and biotechnological applications of fungal EVs. Fungal EVs transport diverse bioactive cargo that collectively contribute to their biological and translational potential. (A) Diagnostic biomarkers: EV-associated proteins, glycans, lipids, and RNAs can be detected in biological fluids such as blood, cerebrospinal fluid (CSF), and urine, providing species-specific signatures for noninvasive fungal diagnostics. Representative biomarker classes include GXM, mannoproteins (Mp88 and Cda1), allergens, miRNA-like molecules, tRNAs, and fungal lipids. Detection approaches include ELISA, qPCR, next-generation sequencing (NGS), and mass spectrometry. (B) Vaccines and immunotherapy: fungal EVs carry immunogenic cargo capable of stimulating innate and adaptive immune responses, including cytokine production, antigen presentation, and Th1/Th17 polarization. Major EV-associated antigens include mannoproteins, glycans, enzymes, and capsular polysaccharides such as GXM. Experimental studies have demonstrated protective immune responses in animal models of candidiasis, cryptococcosis, and aspergillosis. (C) Therapeutic targeting: EV biogenesis and cargo trafficking pathways, including ESCRT machinery, GRASP proteins, SNARE-mediated trafficking, PM budding and autophagy-associated mechanisms, represent potential antifungal targets. Inhibition of EV production or release may impair virulence factor export, biofilm formation, antifungal resistance, and intracellular fungal proliferation. (D) Drug delivery and other applications: the intrinsic cargo transport capacity of fungal EVs supports their potential application as nanoscale delivery systems for drugs, RNAs, and immunomodulatory molecules, including transport across biological barriers such as the BBB. Beyond pathogenic fungi, EVs from environmental and industrial fungi contribute to enzyme delivery, biomass degradation, biofuel production, and bioremediation.

**Table 1.** Extracellular vesicles from representative human pathogenic fungi and their characteristics

Species	First report	Pathogen type (infection site)	Key EV cargo	Key references
*Cryptococcus neoformans*	2007	Opportunistic capsular yeast (Lung, CNS)	GXM, mannoprotein fibrillar coat, SUR7/PalI-family proteins, glyoxal oxidases, chitin deacetylases (CDAs), glucan-remodeling enzymes, virulence-associated enzymes, and immunogenic surface antigens	Mota et al. (2025); Rizzo et al. (2021); Rodrigues et al. (2007, 2008)
*Candida albicans*	2008	Commensal and opportunistic yeast (Mucosal surfaces, blood stream)	Cell wall-remodeling enzymes, Sur7 family proteins, Cdr1/Cdr2 drug-efflux pumps, Plb3 phospholipase, Prd1 proteinase, matrix glucan, and mannan	Albuquerque et al. (2008); Dawson et al. (2020); Gil-bona et al. (2015); Vargas et al. (2015); Wolf et al. (2015); Zarnowski et al. (2018)
*Histoplasma capsulatum*	2008	Dimorphic fungus (Lung)	Superoxide dismutase (SOD), catalase B, glucanases, endochitinases, stress-response RNAs, and lipid-remodeling cargo	Albuquerque et al. (2008); Alves et al. (2019); Zamith-Miranda et al. (2021b)
*Paracoccidioides brasiliensis*	2011	Dimorphic fungus (Lung)	Polysaccharides, α-galactopyranosyl epitopes, lipid species, and regulatory small RNAs	Peres da Silva et al. (2015a, 2015b, 2019); Vallejo et al. (2012)
*Malassezia sympodialis*	2011	Lipophilic commensal yeast (Skin)	Allergens, small RNAs, and inflammatory mediators that induce keratinocyte adhesion molecules and cytokine signaling	Gehrmann et al. (2011); Vallhov et al. (2020)
*Cryptococcus gattii*	2018	Capsular yeast (Lung, CNS)	SUR7/PalI-family proteins, CDA-family proteins, glyoxal oxidases, ferrioxidases, and immunogenic antigens associated with vaccine potential	Bielska et al. (2018); Rizzo et al. (2021)
*Sporothrix brasiliensis*	2018	Dimorphic fungus (Skin)	Immunomodulatory molecules	Ikeda et al. (2018)
*Aspergillus fumigatus*	2019	Filamentous opportunistic fungus (Lung)	Lipid metabolism proteins, cell wall-biosynthesis enzymes, and pathogenicity-associated proteins	Rizzo et al. (2020); Souza et al. (2019)
*Candidozyma auris*	2021	Emerging multidrug-resistant yeast (Blood stream)	Adhesion proteins, neutral lipids, small RNAs, and antifungal resistance-associated cargo	Chan et al. (2022); Zamith-Miranda et al. (2021a)
*Fonsecaea pedrosoi*	2022	Dimorphic fungus (Skin)	Sterol and immunomodulatory molecules	Las-Casas et al. (2022)
*Fonsecaea nubica*	2022	Dimorphic fungus (Skin)	Sterol and immunomodulatory molecules	Las-Casas et al. (2022)
*Candidozyma haemulonii*	2025	Emerging multidrug-resistant yeast (Blood stream)	Proteasome components, glycolytic enzymes, fatty acid metabolism proteins, and miRNA-like molecules	Oliveira et al. (2025)

**Table 2.** Translational applications of fungal extracellular vesicles: Evidence levels and key limitations

Application	Species	Evidence level	Key outcomes	Main limitations	Key references
Diagnostic biomarkers	*C. neoformans*	Patient sera/in vitro	EVs recognized by patient sera; enriched disease-relevant antigens support biomarker potential	Not validated in clinical diagnostic setting; sensitivity/specificity vs. existing assays unknown	Mota et al. (2025); Rizzo et al. (2021); Rodrigues et al. (2008); Wolf et al. (2014)
	*M. sympodialis*	In vitro	EV allergens proposed as biomarkers for atopic dermatitis	Proof-of-concept only; clinical validation pending	Johansson et al. (2018); Vallhov et al. (2020)
	Multiple species	In vitro	Species-specific EV RNA profiles identified; selective enrichment supports discrimination potential	Highly exploratory; stability in patient fluids uncharacterized	Munhoz da Rocha et al. (2021); Peres da Silva et al. (2015b)
Vaccine/Immunotherapy	*C. albicans*	Mouse model	EV immunization induces IgG, reduces fungal burden and confers protection; adjuvant use shifts cytokine profile	Murine model only; optimal antigen/adjuvant formulation undefined; immunization schedule not standardized	Vargas et al. (2015, 2020)
	*C. neoformans*	Mouse model	EV immunization prolongs survival; acapsular mutant-derived EVs confer better protection, suggesting EV surface composition shapes vaccine efficacy	Mechanistic basis of protection not fully defined; no patient-derived validation	Rizzo et al. (2021)
	*Aspergillus* spp.	Mouse model/*G. mellonella*	EV pretreatment improves survival in mouse and *G. mellonella* infection models	Protective antigen identity incompletely defined; limited mechanism and dose-response data	Brauer et al. (2020); Colombo et al. (2020); Souza et al. (2019)
	*P. brasiliensis*	Mouse model	EVs induce protective Th1-mediated response in murine infection models	Single pathogen model; cross-protection against related species not assessed	Vallejo et al. (2012)
	*T. marneffei*	In vitro	EVs induce inflammatory signaling associated with downstream T-cell activation	*In vivo* data not reported yet	Yang et al. (2021)
Therapeutic target	*C. gattii*	In vitro	Hypervirulent strain EVs trigger intracellular proliferation of less virulent strains	No therapeutic target defined so far; in vivo relevance untested	Bielska et al. (2018)
Drug delivery vehicles	Cross-species	Conceptual/early in vitro	Natural barrier crossing properties proposed as basis for delivery	Fungal EV engineering at very early stage; no demonstrated target delivery; *in vivo* safety not assessed	Cai et al. (2018); Huang et al. (2012); van Niel et al. (2018)
